# Prioritizing next-day readiness decline in elite female soccer using readiness, workload, and wellness data: A temporally validated and calibrated monitoring framework

**DOI:** 10.1371/journal.pone.0358036

**Published:** 2026-09-10

**Authors:** Haojie Long, Longji Li, Lifeng Zhang

**Affiliations:** Chengdu Sport University, Chengdu, China; University of Montenegro: Univerzitet Crne Gore, MONTENEGRO

## Abstract

Daily athlete monitoring is widely used in elite soccer, but its value depends on whether routine data can help identify athlete-days more likely to be followed by self-reported next-day readiness decline. This retrospective study evaluated a temporally validated and calibrated model for prioritizing staff-review attention in elite female soccer. Using SoccerMon data, we analyzed 14,428 eligible athlete-days from 50 players. The outcome was same-athlete next-calendar-day self-reported readiness decline ≥0.5 athlete-specific SD calculated from at least five observed readiness values strictly before the index date. Models were trained in 2020 and evaluated in a held-out 2021 test set. Adding workload and wellness to readiness improved discrimination (Model 4 vs Model 0: AUC 0.7645 vs 0.7043; PR-AUC 0.5135 vs 0.3808); paired athlete-level bootstrap ΔAUC was 0.0592 (95% CI 0.0288–0.0891). Platt recalibration reduced Brier score from 0.1924 to 0.1460 without changing ranking. Reviewing the top 20% (1,672 athlete-days) captured 881 events (46.1%) versus 36.4% for Model 0. These retrospective results support cautious staff-review prioritization rather than demonstrated improvement in athlete management or performance, and prospective implementation testing and independent external validation are required before real-world use.

## Introduction

Daily athlete monitoring combines information about what an athlete reports, how they are recovering, and what training exposure they have recently completed. In elite soccer, these data support recovery management, short-term training decisions, and staff workflows because readiness, workload, and wellness measures are low-burden, repeatable, and available close to daily practice. Readiness summarizes the athlete’s current perceived state, wellness items capture perceived fatigue, soreness, sleep, mood, and stress, and workload summaries place those self-reports in the context of recent training exposure [1–5]. However, the methodological and practical challenge is not simply to collect more monitoring data, but to evaluate whether repeated daily reports can be converted into review priorities that staff can interpret before the next training decision.

Load-wellness relationships are heterogeneous and context dependent. Female team-sport athletes can move through congested match periods, travel, variable microcycles, changing academic or professional obligations, and different recovery opportunities. A similar workload can therefore carry different meaning depending on timing, training plan, sleep, prior soreness, and the reporting culture within a team. Recent soccer and female team-sport research has reinforced the need to interpret training load, perceived exertion, sleep, GPS-derived variables, and wellness questionnaires within sport-, team-, and season-specific contexts rather than as universal stand-alone signals [6–12].

Machine-learning and statistical prediction approaches have increasingly been applied to perceived wellness, readiness, fatigue, and athlete health monitoring. Prior soccer studies have shown that preceding workload and wellness information can forecast perceived wellness or readiness-related states [13–16]. Related sport applications have also used machine-learning models for fatigue, athlete monitoring, health, recovery, and injury-related endpoints [17–20]. Injury-forecasting studies, for example, address a different endpoint from next-day readiness decline; their methodological lessons are useful, but their clinical or injury-risk framing should not be transferred directly to a self-reported monitoring outcome [21].

Several methodological issues are especially important for repeated athlete-day data. Random row-level splitting can place observations from the same athlete, team, or season context in both training and testing data, producing optimistic estimates when the practical question is whether information available at one time supports interpretation of future athlete states. Previous soccer monitoring and machine-learning studies also show that workload, readiness, and monitoring relationships can vary across models, seasons, and microcycle contexts [13,15,20,22,23]. Repeated observations within athletes therefore make uncertainty estimates and threshold performance sensitive to clustering, missingness, and athlete-specific variability.

Discrimination, threshold performance, and calibration answer distinct questions. AUC and PR-AUC describe how well a model ranks athlete-days, operating thresholds determine sensitivity, specificity, F1, and predicted-positive burden, and calibration evaluates whether predicted probabilities match observed event frequencies. A model may rank athlete-days usefully while assigning probabilities that are systematically too high or too low. This distinction is particularly important when class-weighted models are used to address event imbalance and when probabilities could influence staff attention or athlete communication.

Using the SoccerMon dataset [24], this study examined whether routinely collected readiness, workload, and wellness indicators could improve the prioritization of athlete-days followed by next-day readiness decline. The next-day horizon was selected because it corresponds to the subsequent monitoring cycle and is directly relevant to short-term staff review. The primary objective was to evaluate whether adding workload and wellness domains improved discrimination beyond current readiness alone in a chronologically held-out season. Secondary objectives were to assess operating-threshold performance, probability calibration, recalibration, practical top-risk capture, athlete-grouped robustness, cross-team robustness, and sensitivity to alternative athlete-specific readiness-SD definitions. The analysis was positioned as a retrospective prediction-model evaluation for a self-reported monitoring outcome, not as causal inference, injury prediction, or an automated decision system.

The central analytical expectation was that adding workload and wellness information would provide incremental predictive information beyond current readiness alone.

## Materials and methods

### Study design and data source

This was a retrospective secondary analysis of public SoccerMon subjective monitoring data from 2020 and 2021 [24]. The panel contained 36,550 rows from 50 elite female soccer players from two teams. All players provided written informed consent to participate, including open publication of recorded data. The original SoccerMon study was approved by the Norwegian Privacy Data Protection Authority (reference 296155) and was exempted from approval by the Regional Committee for Medical and Health Research Ethics – South East Norway and the Regional Committee for Medical and Health Research Ethics – Northern Norway. The publicly released dataset was de-identified. It was accessed on 20 May 2026; the authors had no access to identifiable participant information. This secondary analysis involved no new recruitment, intervention, data collection, participant contact, or additional consent.

The analysis was designed to evaluate whether monitoring information available on an index day could support interpretation of next-calendar-day readiness decline. Repeated athlete-days were treated as repeated observations nested within athletes, not as independent athletes. The intended use case was staff review of a short-term self-reported monitoring outcome, not causal inference, clinical diagnosis, injury prediction, automated prescription, or direct training-plan modification.

### Participants and daily monitoring data

The dataset included 50 elite female soccer players from two teams. Daily monitoring variables included self-reported readiness and wellness dimensions, together with training-load summaries derived from session-RPE and related workload calculations. Observations spanned the 2020 and 2021 monitoring years. Because the data were public and de-identified, the analysis used the available variables as recorded and did not attempt to infer unobserved contextual factors such as match congestion, travel, menstrual-cycle status, injuries, illness, tactical load, or staff decisions.

The primary analysis used athlete-day rows only when the strict next-day outcome could be observed for the same athlete. Daily rows remained repeated measures; therefore, chronological validation, athlete-grouped out-of-fold threshold selection, athlete-level bootstrap resampling, and cross-team robustness checks were used to reduce overinterpretation of row-level performance estimates.

### Outcome eligibility and definition

Outcome eligibility required a valid athlete identifier and date, observed index-day readiness, observed next-calendar-day readiness for the same athlete, and at least five prior observed readiness values. Of 36,550 daily rows, 14,428 (39.5%) were eligible; missing index-day readiness was the dominant exclusion and unavailable next-day readiness the next major exclusion. Descriptively, eligibility was 33.2% in 2020 and 45.8% in 2021, and 48.7% in Team A versus 28.7% in Team B; athlete-level and predictor-level completeness varied and are summarized in the Supplementary Material. Missingness mechanisms could not be established and may reflect availability, participation, reporting behavior, or team monitoring practice. Outcomes were not imputed.

Of the 36,550 source daily rows, 2,355 (6.4%) were excluded because same-athlete next-day readiness was unavailable. Final eligibility was 33.2% in 2020 and 45.8% in 2021, and 48.7% in Team A versus 28.7% in Team B. Across the 50 athletes, final eligibility ranged from 0.4% to 86.7%; among rows with an observed index-day readiness and a same-athlete next-calendar-day record, next-day readiness unavailability ranged from 0.9% to 78.3% (median 15.6%). These are descriptive differences only and do not establish a missingness mechanism. Full sequential and athlete-level summaries are provided in Supplementary Table S14 in S1 File; predictor missingness and monitoring completeness are provided in Supplementary Table S2 in S1 File.

The primary event was same-athlete next-calendar-day self-reported readiness decline ≥0.5 athlete-specific SD from index-day readiness. For each index date, SD was calculated only from observed readiness values strictly before the index date, requiring at least five prior observations (expanding-history min5). Index-day readiness, next-day readiness, and all future values were excluded from the SD history. The 0.5-SD threshold was an athlete-relative, distribution-based operational definition, not a clinically validated or physiologically established cutoff. The use of 0.5 SD has precedent as a distribution-based benchmark for interpreting change in self-reported outcomes [25,26], but distribution-based evidence alone does not establish clinical importance or performance relevance.

Sensitivity analyses evaluated 0.25-, 0.50-, 0.75-, and 1.00-SD definitions and alternative history windows. Expanding-history min5 was primary. The full-period 2020–2021 SD definition was retained only as a retrospective sensitivity/reference analysis; fixed-2020 and expanding-history min2 and min7 were also sensitivity analyses.

Detailed results for the alternative readiness-decline magnitudes are provided in Supplementary Table S15 in S1 File.

### Predictor construction and model sets

Predictors were organized into five nested model sets. Model 0 included index-day readiness only (1 predictor). Model 1 added workload magnitude variables (15 predictors). Model 2 added workload-variability features (23 predictors), including the 14-day rolling standard deviation of daily load; these predictors were retained in Models 3 and 4. Model 3 added workload instability variables (25 predictors). Model 4 added wellness features and contained 55 predictors. The complete ordered predictor list is provided in Supplementary Table S1 in S1 File.

The model hierarchy was intended to quantify incremental predictive information from readiness, workload magnitude, workload variability, workload instability, and wellness domains. All predictor sets were prespecified from the available SoccerMon monitoring variables and were used consistently for training and validation matrices. Predictor coefficients were treated as model-dependent predictive associations rather than causal effects.

### Missing-data handling and preprocessing

No outcome imputation was performed. Predictor preprocessing was fitted inside the relevant training data only. Median imputation was fitted on training rows and then applied to validation or test rows. For logistic regression, predictors were standardized after imputation using training-data scaling parameters. The same imputation and scaling discipline was used for chronological validation and athlete-grouped out-of-fold threshold selection. Predictor missingness and athlete-level monitoring completeness are summarized in Supplementary Table S2 in S1 File. Detailed analytical-set characteristics across the alternative athlete-specific readiness-SD definitions are provided in Supplementary Table S3 in S1 File.

The 2021 test set was not used for fitting imputation parameters, scaling parameters, model coefficients, feature selection, operating thresholds, or recalibration models. This preserved the chronological test set as an untouched held-out validation period for the primary analysis.

### Model development

The primary logistic model used L2 penalty, lbfgs solver, C = 1.0, fit_intercept = true, class_weight = “balanced,” max_iter = 5000, and random_state = 42. C = 1.0 was prespecified, not optimal. The 14,428 athlete-days are repeated observations from only 50 athletes and two teams; complexity control used L2 regularization, nested predictor sets, chronological evaluation, athlete-grouped OOF procedures, and athlete-level bootstrap. Performance was materially stable across C = 0.1–3.0. Balanced class weighting reduced majority-class dominance but raw weighted-model probabilities were not assumed calibrated.

A random-forest comparator was fitted with n_estimators = 250, max_depth = 5, min_samples_split = 2, min_samples_leaf = 10, max_features = “sqrt,” bootstrap = true, class_weight = “balanced,” random_state = 42, and n_jobs = −1. The random forest was used as a nonlinear comparator and did not replace the logistic-regression primary analysis. The shallow depth and minimum leaf size were used as an intentionally regularized comparator specification to limit tree complexity given the limited athlete-level sample; a restricted sensitivity analysis with deeper and larger forests was examined separately without using 2021 labels for hyperparameter selection.

Detailed athlete-grouped validation is provided in Supplementary Table S8 in S1 File; regularization sensitivity and leading coefficient-direction stability are provided in Supplementary Table S16 in S1 File; the random-forest comparator sensitivity is provided in Supplementary Table S17 in S1 File.

### Temporal and robustness validation

Primary validation trained models on 2020 athlete-days and tested them on 2021 athlete-days. The 2021 test set was left untouched until final evaluation. Threshold-independent metrics included AUC, PR-AUC, and Brier score. Threshold-dependent metrics included sensitivity, specificity, balanced accuracy, F1, and predicted-positive rate.

Robustness analyses included five-fold athlete-grouped validation and internal cross-team holdout checks. The athlete-grouped analysis kept all rows from a given athlete in the same fold. The cross-team holdout trained on one SoccerMon team and tested on the other, then repeated the reverse direction. This within-dataset cross-team analysis was interpreted as robustness evidence only and was not described as validation in an independent cohort.

### Operating-threshold selection

The primary threshold was selected using expanding-history min5 2020 athlete-grouped OOF predictions, balanced-accuracy optimization, and no 2021 test labels. The selected threshold was 0.501 (0.5011351162) and was applied unchanged to 2021 predictions. Detailed threshold-selection results are provided in Supplementary Table S4 in S1 File.

The older training-apparent balanced-accuracy threshold was retained only as a historical comparison of threshold selection procedures and was not used as the primary classification result. The manuscript therefore reports the athlete-grouped out-of-fold threshold as the main operating threshold.

### Calibration and recalibration

Calibration was assessed on 2021 predictions using observed prevalence, mean predicted probability, mean prediction error, Brier score, constant-risk Brier reference, Brier Skill Score, calibration-in-the-large from an offset logistic model, calibration intercept and slope from a freely estimated intercept-and-slope logistic model, expected calibration error, maximum calibration error, and reliability-bin summaries. Detailed calibration metrics and reliability-bin summaries are provided in Supplementary Table S5 and Supplementary Table S6 in S1 File.

Unweighted Platt recalibration was fitted only on 2020 athlete-grouped OOF predictions and applied once to 2021 predictions. It was a monotonic low-complexity parametric recalibration chosen to limit flexibility given limited athlete-level diversity. It improved probability alignment without changing AUC or PR-AUC. Platt scaling was preferred to more flexible non-parametric calibration such as isotonic regression to limit recalibration complexity in the available athlete-grouped OOF sample.

### Practical prioritization analysis

To evaluate practical staff-review prioritization, 2021 athlete-days were ranked from highest to lowest predicted probability of next-day readiness decline using the primary Model 4 logistic-regression scores. Platt-recalibrated probabilities were used for probability-level summaries. Because the fitted Platt transformation was monotonic, ranking-based top-risk capture metrics were unchanged by recalibration. At prespecified review proportions of 5%, 10%, 20%, 30%, 40%, and 50%, we calculated the number of athlete-days reviewed, the number and proportion of readiness-decline events captured, precision among reviewed athlete-days, lift relative to the 2021 event prevalence, and athlete-days reviewed per captured event. The same analysis was repeated for the readiness-only Model 0, with a no-skill diagonal reference included in cumulative-gains plots.

### Statistical uncertainty and software

Uncertainty used 1,000 athlete-level bootstrap replicates, resampling athletes rather than rows. For paired incremental comparisons, Model 0 and Model 4 were evaluated on the same sampled athletes within every replicate; percentile 95% CIs were reported. Detailed athlete-level bootstrap estimates are provided in Supplementary Table S7 in S1 File.

Analyses used Python 3.12.3 with pandas 3.0.3, NumPy 2.4.6, SciPy 1.17.1, scikit-learn 1.8.0, statsmodels 0.14.6, matplotlib 3.10.9, and joblib 1.5.3 for data handling, model development, validation, calibration, uncertainty estimation, and figure generation.

## Results

### Sample construction

**Fig 1:**
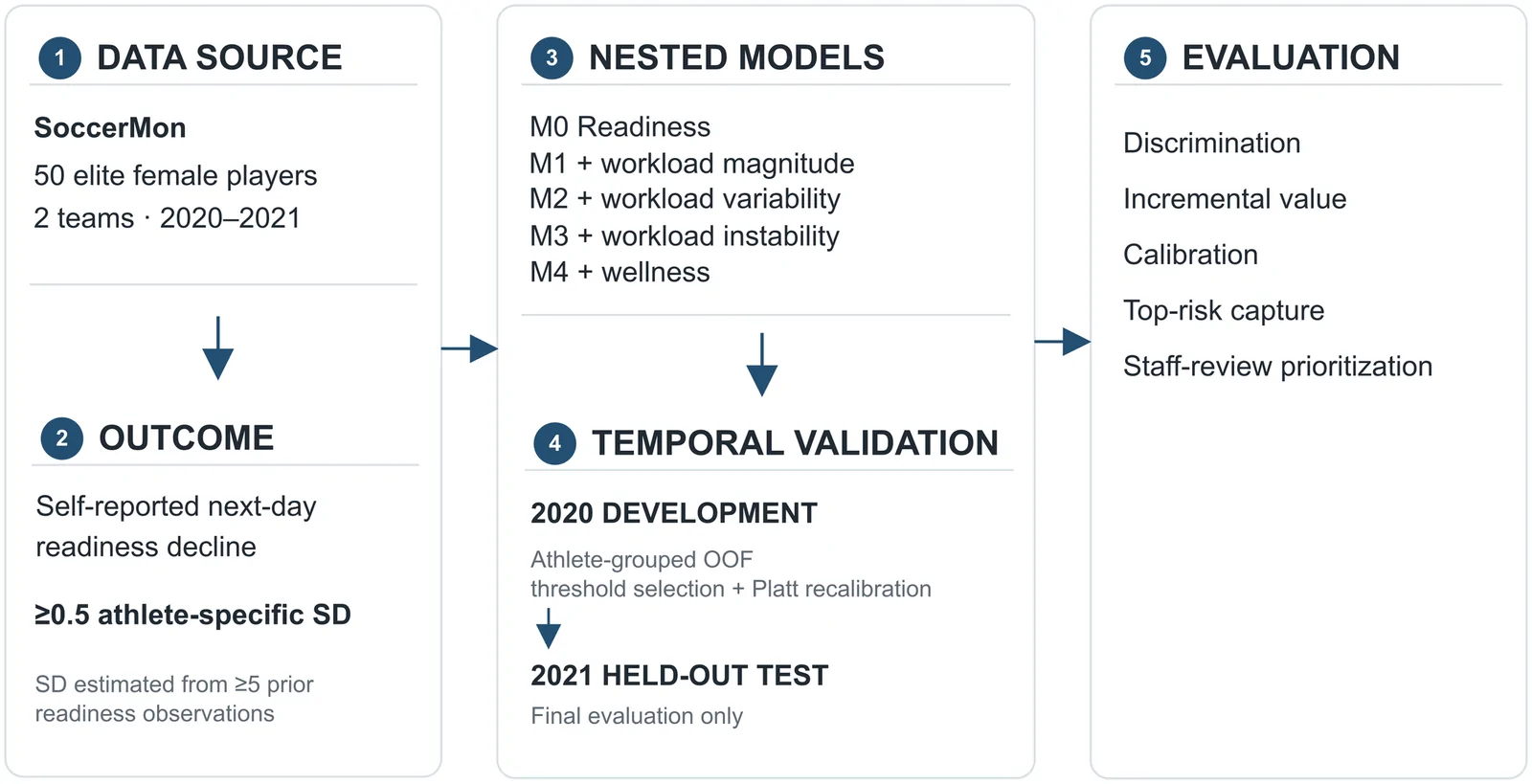
Study workflow and validation design. The workflow summarizes the temporally valid expanding-history min5 outcome, 2020 development, athlete-grouped OOF threshold selection, Platt recalibration, and 2021 held-out evaluation.

Sample construction and the analytical workflow are summarized in Table 1 and Fig 1. The expanding-history min5 primary dataset contained 14,428 athlete-days and 3,390 events (23.5%). The 2020 development set contained 6,072 athlete-days and 1,479 events; the 2021 held-out test set contained 8,356 athlete-days and 1,911 events.

**Table 1:** Sample construction and chronological analysis sets.

Analysis set	Observation period	Athletes	Athlete-days	Readiness-decline events	Event rate	Analytical role
Complete daily panel	2020–2021	50	36,550	—	—	Source monitoring panel
Strict observed-pair primary dataset	2020–2021	50	14,428	3,390	0.235	Expanding-history min5 primary outcome dataset
2020 training dataset	2020	50	6,072	1,479	0.244	Model fitting, preprocessing, threshold selection, and recalibration fitting
2021 held-out test dataset	2021	50	8,356	1,911	0.229	Untouched chronological test period

Eligibility required same-athlete next-calendar-day readiness after an observed index-day readiness value; outcomes were not imputed. For the primary expanding-history min5 outcome, athlete-specific SD was estimated from at least five observed readiness values strictly before each index date. Event rates use readiness-decline events divided by athlete-days in the corresponding analytical set.

These counts reflect the strict observed-pair expanding-history min5 primary outcome requirement. Rows without observed same-athlete next-calendar-day readiness were not labeled as events or non-events. The athlete count remained 50 in the primary analysis.

### Primary chronological validation

**Fig 2:**
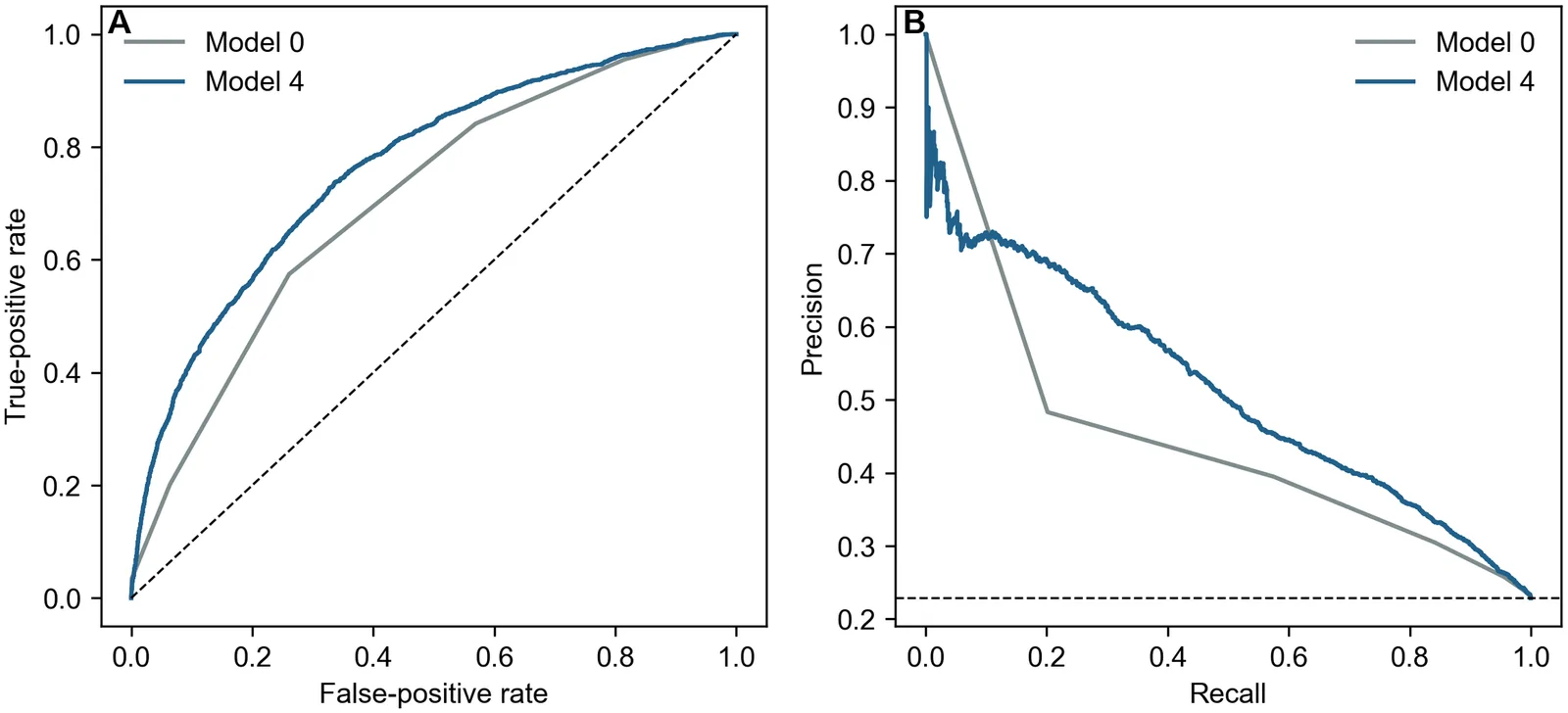
Discrimination of the primary Model 4 in the held-out 2021 test set. Panel A shows the ROC curve, and Panel B shows the precision–recall curve. AUC was 0.764 and PR-AUC was 0.513.

Chronological validation is summarized in Table 2, and the discrimination performance of the primary Model 4 is shown in Fig 2. In 2020-to-2021 validation, primary Model 4 logistic regression achieved AUC 0.7645, PR-AUC 0.5135, and Brier 0.1924, compared with Model 0 AUC 0.7043, PR-AUC 0.3808, and Brier 0.2206. Paired athlete-level bootstrap showed ΔAUC 0.0592 (95% CI 0.0288–0.0891), ΔPR-AUC 0.1296 (95% CI 0.0897–0.1677), and Brier improvement 0.0281 (95% CI 0.0141–0.0435), indicating incremental predictive information rather than perfect individual prediction.

**Table 2:** Chronological validation of nested prediction models in the 2021 test set.

Model	Predictor domains	Number of predictors	Algorithm	AUC	PR-AUC	Brier score
Model 0	Index-day readiness	1	Logistic regression	0.704	0.381	0.221
Model 1	+ workload magnitude	15	Logistic regression	0.748	0.494	0.205
Model 2	+ workload variability	23	Logistic regression	0.754	0.499	0.207
Model 3	+ workload instability	25	Logistic regression	0.753	0.497	0.207
Model 4	+ wellness	55	Logistic regression	0.764	0.513	0.192
Model 4	+ wellness	55	Random forest	0.747	0.487	0.203

AUC denotes area under the receiver operating characteristic curve; PR-AUC denotes area under the precision–recall curve. Brier score measures mean squared probability error. All models were trained on 2020 athlete-days and evaluated in the untouched 2021 test period. Model 4 logistic regression was the primary model; random forest was a nonlinear comparator.

The largest absolute standardized coefficients in the primary Model 4 included weekly load, acute training load (ATL), current readiness, and daily load. Coefficient direction was less stable for weekly load than for several other leading predictors across athlete-grouped folds. These coefficients were therefore interpreted as model-dependent coefficient summaries rather than causal or independently actionable measures of variable importance. Detailed standardized coefficients and fold-level stability results are provided in the Supplementary Material.

Supplementary Table S11 in S1 File presents the standardized coefficient ranking, and Supplementary Table S16 in S1 File presents the leading coefficient-direction stability results.

The nested model sequence showed incremental gains when workload and wellness domains were added. Logistic-regression AUCs were 0.704 for Model 0, 0.748 for Model 1, 0.754 for Model 2, 0.753 for Model 3, and 0.764 for Model 4. The Model 4 random-forest comparator had AUC 0.747 and PR-AUC 0.487, which did not exceed the primary logistic-regression model in chronological testing.

### Operating-threshold performance

Operating-threshold performance used the 2020 athlete-grouped OOF threshold of 0.501. Held-out 2021 sensitivity was 0.687, specificity 0.704, balanced accuracy 0.695, F1 0.512, and predicted-positive rate 0.386. The corresponding AUC, PR-AUC, and Brier were 0.7645, 0.5135, and 0.1924.

An older apparent-training thresholding procedure was reviewed only as a historical comparison and was not treated as the primary operating-threshold result. The out-of-fold threshold was therefore used for the principal threshold-dependent interpretation.

### SD-definition sensitivity

Sensitivity to athlete-specific readiness-SD definition is summarized in Table 3. Expanding-history min5 is the primary definition. Full-period SD is retrospective sensitivity/reference only; fixed-2020 SD and expanding-history min2 and min7 are sensitivity analyses.

**Table 3:** Sensitivity of primary Model 4 performance to athlete-specific readiness-SD definition.

SD definition	Athletes	Eligible athlete-days	Events	AUC	PR-AUC	Brier score
Expanding-history min5 (primary)	50	14,428	3,390	0.764	0.513	0.192
Full-period SD (retrospective sensitivity/reference)	50	14,631	3,462	0.766	0.521	0.190
Fixed-2020 SD (sensitivity)	37	13,231	3,114	0.768	0.521	0.183
Expanding-history min2 (sensitivity)	50	14,516	3,420	0.764	0.513	0.193
Expanding-history min7 (sensitivity)	50	14,347	3,362	0.763	0.511	0.193

Expanding-history min5 was the primary analysis. Full-period SD was retained only as retrospective sensitivity/reference; fixed-2020 SD and expanding-history min2 and min7 were sensitivity analyses. Full-period SD used all observed 2020–2021 readiness values for each athlete before strict-pair filtering. Fixed-2020 SD used observed 2020 readiness values. Expanding-history SD used only readiness observations strictly before each index date, excluding index-day and future readiness, with minimum prior-history requirements of 2, 5, or 7 observations.

The primary expanding-history min5 analysis retained 14,428 rows from 50 athletes, with 3,390 events. It produced AUC 0.764, PR-AUC 0.513, and Brier score 0.192.

Secondary expanding-history checks were similar: min2 retained 14,516 rows and achieved AUC 0.764, whereas min7 retained 14,347 rows and achieved AUC 0.763. These analyses supported the robustness of the primary finding while making clear that full-period SD was a retrospective outcome-definition choice.

Results were also robust to the magnitude used to define readiness decline: across 0.25-, 0.50-, 0.75-, and 1.00-SD event thresholds, Model 4 consistently exceeded Model 0, with ΔAUC ranging from 0.0504 to 0.0608 and ΔPR-AUC from 0.0967 to 0.1403. This sensitivity supports robustness of the incremental-prediction finding to the operational cutoff rather than establishing clinical meaning for 0.5 SD.

### Athlete-grouped and cross-team robustness

In the expanding-history min5 robustness analysis, five-fold athlete-grouped Model 4 logistic validation averaged AUC 0.766, PR-AUC 0.530, balanced accuracy 0.686, sensitivity 0.668, specificity 0.705, F1 0.509, predicted-positive rate 0.383, and Brier score 0.200 across 5 folds. Fold-level athlete-grouped robustness results are provided in Supplementary Table S8 in S1 File.

Internal cross-team holdout checks within SoccerMon produced Model 4 logistic AUCs of 0.757 for training on TeamA and testing on TeamB, and 0.748 for training on TeamB and testing on TeamA. Mean cross-team holdout AUC was 0.753 and mean PR-AUC was 0.487. These checks should be read as within-dataset robustness analyses, not validation in an independent cohort. The within-SoccerMon cross-team holdout robustness analysis is provided in Supplementary Table S9 in S1 File.

### Calibration and recalibration

**Fig 3:**
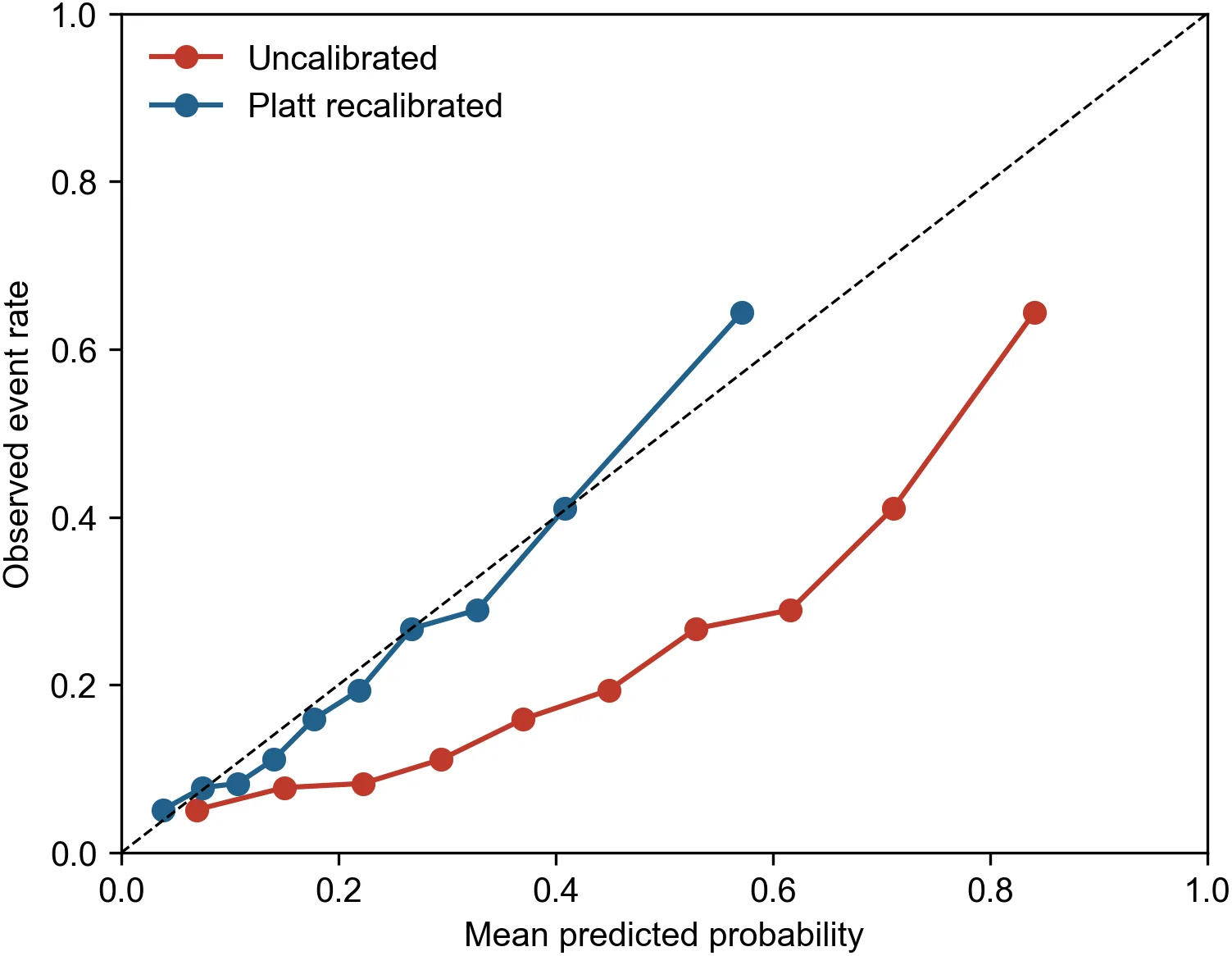
Calibration of the primary expanding-history min5 Model 4 before and after Platt recalibration in the held-out 2021 test set.

Calibration performance is summarized in Table 4, and reliability curves are shown in Fig 3. In the 2021 test set, observed prevalence was 0.2287, whereas the uncalibrated mean predicted probability was 0.4254. The uncalibrated Brier score was 0.1924 compared with a constant-risk reference of 0.1764, yielding a Brier Skill Score of −0.0905. Uncalibrated calibration-in-the-large was −1.1686, calibration intercept was −1.1493, calibration slope was 0.9002, ECE was 0.1967, and MCE was 0.3268.

**Table 4:** Operating-threshold and probability-calibration performance of the primary Model 4 logistic regression in the 2021 test set.

Panel A. Primary grouped-OOF operating threshold						
Threshold source	Threshold	Sensitivity	Specificity	Balanced accuracy	F1	Predicted-positive rate
2020 athlete-grouped OOF predictions	0.501	0.687	0.704	0.695	0.512	0.386
**Panel B. Probability calibration**						
**Metric**	**Uncalibrated**	**Platt recalibrated**				
Observed prevalence	0.229	0.229				
Mean predicted probability	0.425	0.233				
Brier score	0.192	0.146				
Constant-risk Brier reference	0.176	0.176				
Brier Skill Score	−0.090	0.173				
Calibration-in-the-large	−1.169	−0.031				
Calibration intercept	−1.149	0.077				
Calibration slope	0.900	1.110				
Expected calibration error	0.197	0.023				
Maximum calibration error	0.327	0.072				

The operating threshold was selected using five-fold athlete-grouped out-of-fold (OOF) predictions from 2020 and applied unchanged to 2021. Platt recalibration was fitted without class weighting using only 2020 grouped-OOF predictions and applied once to untouched 2021 predictions. AUC and PR-AUC were unchanged by monotonic recalibration. Calibration-in-the-large came from an offset model; calibration intercept and slope came from a freely estimated model.

After unweighted Platt recalibration fitted only on 2020 athlete-grouped out-of-fold predictions, the 2021 mean predicted probability was 0.2334, Brier score 0.1460, and Brier Skill Score 0.1725. Recalibrated calibration-in-the-large was −0.0307, calibration intercept 0.0765, calibration slope 1.1098, ECE 0.0226, and MCE 0.0721. Recalibration improved probability alignment but did not change ranking metrics.

### Practical prioritization performance

**Fig 4:**
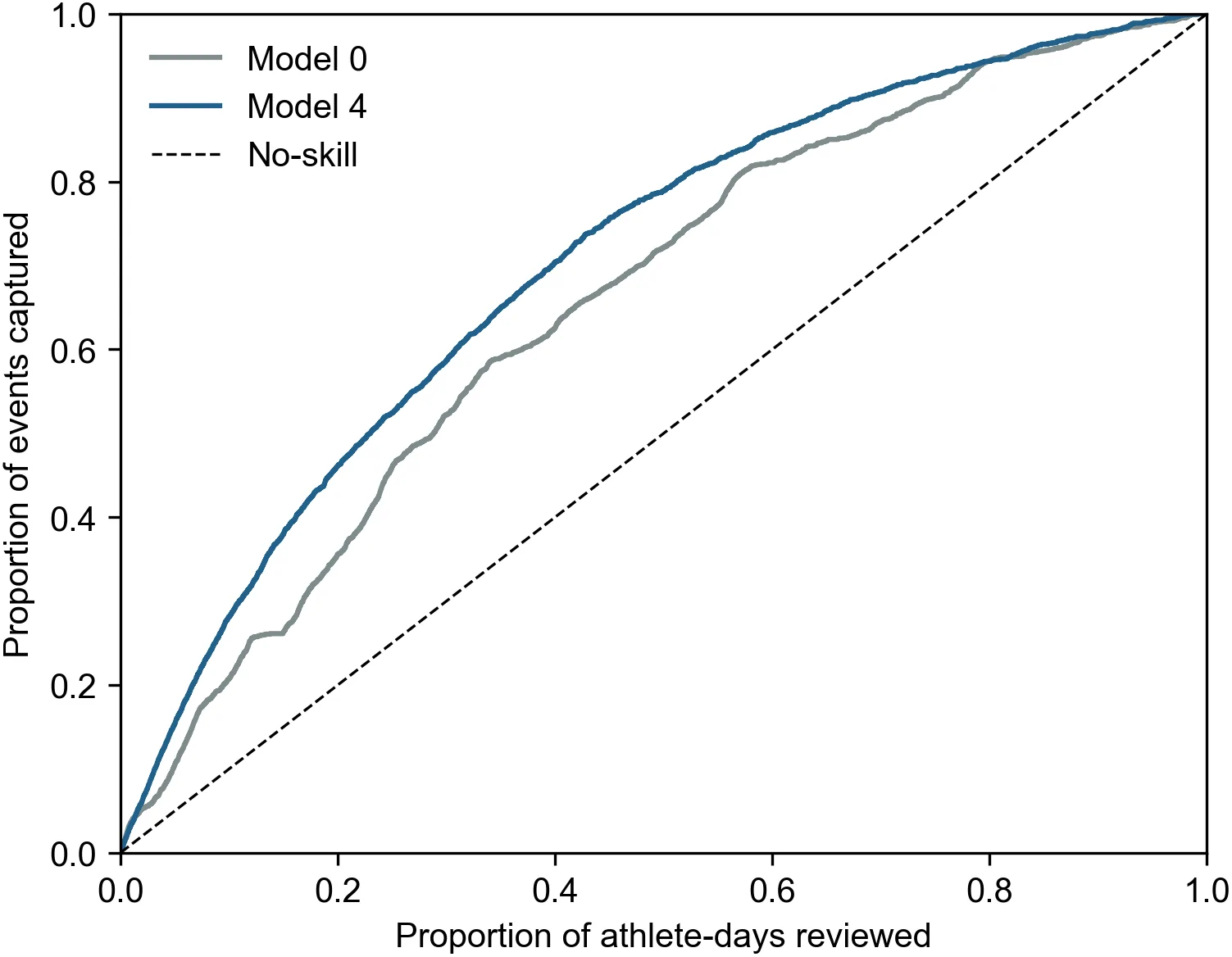
Cumulative gains for top-risk staff-review prioritization in the primary expanding-history min5 analysis. Athlete-days were ranked by predicted probability of next-day self-reported readiness decline. Model 4 is compared with the readiness-only Model 0 and the no-skill reference.

The practical prioritization analysis showed that the model concentrated readiness-decline events in the highest-ranked athlete-days (Table 5 and Fig 4). Table 5 presents all primary min5 top-risk rows from 5% to 50%. At the top 20%, Model 4 reviewed 1,672 athlete-days and captured 881 events (46.1% capture; 52.7% PPV; lift 2.30 relative to the 2021 event prevalence of 22.9%; 1.90 athlete-days per captured event), compared with 36.4% capture for Model 0. This prioritization result does not demonstrate improved staff decisions or athlete outcomes.

**Table 5:** Practical prioritization performance of the primary Model 4 in the 2021 test set.

Review proportion	Athlete-days reviewed	Events captured	Event capture rate	Precision among reviewed athlete-days	Lift versus test prevalence	Athlete-days per event captured
Top 5%	418	295	15.4%	70.6%	3.09	1.42
Top 10%	836	538	28.2%	64.4%	2.81	1.55
Top 20%	1,672	881	46.1%	52.7%	2.30	1.90
Top 30%	2,507	1,123	58.8%	44.8%	1.96	2.23
Top 40%	3,343	1,346	70.4%	40.3%	1.76	2.48
Top 50%	4,178	1,508	78.9%	36.1%	1.58	2.77

Athlete-days were ranked from highest to lowest predicted probability of next-day readiness decline. Event capture rate denotes captured events divided by all readiness-decline events in the 2021 test set. Precision denotes the event rate among reviewed athlete-days. Lift denotes precision divided by the observed 2021 test-set event prevalence. Complete top-risk capture results, including Model 0 comparisons, are provided in Supplementary Table S10 in S1 File.

### Athlete-level bootstrap uncertainty

For the primary analysis, paired athlete-level bootstrap supported Model 4 versus Model 0: ΔAUC 0.0592 (95% CI 0.0288–0.0891), ΔPR-AUC 0.1296 (95% CI 0.0897–0.1677), and Brier improvement 0.0281 (95% CI 0.0141–0.0435), based on 1,000 athlete-level replicates. These are paired incremental uncertainty estimates; they do not demonstrate prospective clinical or decision impact.

## Discussion

### Principal findings

This retrospective study evaluated whether routine readiness, workload, and wellness data could prioritize athlete-days followed by self-reported next-day readiness decline in elite female soccer. The primary outcome used expanding-history min5, whereas the full-period SD definition was retained only as a retrospective sensitivity/reference analysis. Model 4 added incremental predictive information beyond readiness alone. At the top 20% review burden, Model 4 captured approximately 46.1% of events versus 36.4% for Model 0. Platt recalibration improved probability alignment. These findings support prioritization for staff review, not automated training prescription or demonstrated improvement in athlete outcomes.

From an applied perspective, the model should be interpreted as a prioritization layer within an existing monitoring process. The value of the framework is not that it automatically determines training modification, but that it can enrich the subset of athlete-days selected for staff review. Such review should incorporate training plans, recent match demands, athlete comments, travel, injury or illness context, and staff observations. The top-risk capture results are therefore more practically informative than a single operating threshold because they allow staff to select a review burden that matches available resources.

### Comparison with previous research

The study extends descriptive load-wellness research by testing a future self-reported monitoring outcome rather than only contemporaneous associations among load, fatigue, wellness, and performance-related indicators. This temporal framing is important because applied staff questions often concern whether today’s monitoring information helps interpret tomorrow’s athlete state, not whether two variables are associated on the same day. The findings are consistent with the broader view that subjective wellness and internal-load measures can provide useful monitoring information in team sports, while remaining context dependent [1–12].

The analysis also differs from previous machine-learning readiness and wellness studies by emphasizing transparent logistic regression, chronological validation, operating-threshold selection, and calibration. Prior soccer studies have forecast perceived wellness or readiness-related states from preceding load and wellness variables [13–16]. Related sport applications have also used machine-learning models for fatigue, health, recovery, athlete monitoring, or injury-related endpoints [17–21]. The present endpoint was not injury, illness, clinical readiness, or match performance. It was a short-term self-reported readiness-decline label, so comparisons with injury forecasting should remain methodological rather than clinical.

Recent soccer workload studies highlight the complex relationship between internal load, external load, perceived exertion, sleep, technical-tactical context, microcycle structure, and athlete well-being [7–12,22,23]. The current results align with that complexity: workload and wellness domains improved ranking performance, but the model does not identify causal drivers of readiness decline. Correlated predictors, class weighting, team-specific monitoring practices, and repeated reporting culture limit interpretation of individual coefficients as intervention targets.

### Outcome-definition and temporal-validity considerations

The full-period SD definition incorporates post-index readiness observations into the label threshold and therefore cannot be constructed prospectively at the index date. The primary analysis consequently used expanding-history min5, for which SD was calculated only from observed readiness values strictly before the index date, with at least five prior observations. The full-period definition was retained as a retrospective sensitivity/reference analysis. Similar performance across definitions supported robustness to outcome-definition choice.

The 0.5-SD rule should therefore be interpreted only as an athlete-relative, distribution-based operational threshold. It is not clinically validated, physiologically established, or performance-relevant, and the sensitivity analyses show robustness of the incremental predictive finding to the operational cutoff rather than validation of its clinical or performance meaning.

The expanding-history min5 primary analysis addresses this temporal-validity concern while retaining 50 athletes. It remains retrospective and does not constitute prospective validation. A true prospective study would require pre-specified procedures, ongoing data collection, and evaluation in a future or independent team context, consistent with prediction-model validation principles [27–29].

The chronological 2020–2021 split was central to the analysis. Random row splitting would be less appropriate because repeated athlete-day observations can share athlete-specific response patterns, team practices, and seasonal context across rows. The athlete-grouped and cross-team robustness analyses provided additional checks, but the cross-team holdout remained within the SoccerMon dataset and should be described only as an internal robustness check.

### Calibration and practical interpretation

Calibration was a major practical finding and is central to the interpretation of prediction-model outputs [27,28,30]. Raw class-weighted probabilities overestimated absolute risk: the observed 2021 prevalence was approximately 0.229, while the uncalibrated mean predicted probability was approximately 0.425. This discrepancy means that uncalibrated probabilities should not be interpreted as literal event risks, even though the model ranked athlete-days with useful discrimination. Poor raw calibration is plausible because class weighting changes the estimation target and can shift the probability scale.

Unweighted Platt recalibration fitted only on 2020 athlete-grouped out-of-fold predictions improved mean probability alignment, Brier score, Brier Skill Score, and calibration error in the held-out 2021 data. This is encouraging, but recalibration remains sample- and context-dependent. Calibration should be reassessed before any future use in a different team, season, league, country, or monitoring culture. The distinction between ranking and probability interpretation should be explicit whenever model outputs are communicated to practitioners. This makes calibration an applied requirement for staff-facing monitoring tools, not only a statistical reporting detail.

From a practical perspective, a flagged athlete-day should initiate staff review rather than prescribe a training change. Model outputs should be integrated with coaching knowledge, medical context, athlete communication, privacy safeguards, and the limitations of self-report data. Over-reliance on predicted probabilities could harm trust in monitoring systems if athletes perceive scores as surveillance or automatic decision rules.

A numeric predicted probability may also frame staff perceptions of an athlete and influence how subsequent self-reports or contextual information are interpreted; scores should therefore function as prompts for contextual review rather than recommendations for action.

High predicted scores could lead to unnecessary scrutiny or intervention, whereas low predicted scores could create false reassurance; model outputs should therefore remain subordinate to professional judgment and athlete-centered communication. At the illustrative 20% review burden, staff attention would be directed to one fifth of athlete-days while capturing 46.1% of subsequent readiness-decline events, but the present retrospective study did not measure staff time saved, decision quality, or implementation benefit.

### Strengths and limitations

Strengths include use of public SoccerMon data [24], a strict same-athlete next-calendar-day observed-pair outcome, no outcome imputation, a transparent nested model hierarchy, 2020–2021 chronological validation, athlete-grouped out-of-fold threshold selection, calibration analysis, training-only recalibration, athlete-level bootstrap uncertainty, and sensitivity analyses for SD-definition choices. The analysis also explicitly separated discrimination, threshold-dependent performance, calibration, and robustness checks, consistent with reporting principles for prediction models [27,28].

Limitations are substantial. The athlete-level sample contained only 50 players from two teams; therefore, the 14,428 repeated athlete-days do not substitute for athlete-level diversity and should not be interpreted as 14,428 independent participants. The data were secondary and de-identified, so contextual variables, data-collection timing, compliance mechanisms, staff interventions, menstrual-cycle context, injury or illness status, and missingness mechanisms could not be fully evaluated. These unmeasured contextual factors may influence both readiness reporting and workload exposure and could therefore contribute to unexplained variation or shifts in model discrimination and calibration, although the direction of any resulting bias cannot be determined from these data. The outcome was self-reported readiness decline rather than injury, illness, clinical readiness, match performance, or objective recovery. The analysis lacked an independent external cohort and lacked prospective implementation. Generalizability across teams, leagues, countries, competitive levels, and seasons remains uncertain.

Additional limitations arise from the modeling framework. Predictors were correlated, class weighting affected probability calibration, and athlete-specific SD thresholds depend on the selected historical window. The cross-team holdout involved only two teams from the same SoccerMon dataset and should be viewed solely as an internal robustness analysis; it does not constitute independent external validation or establish transportability to other teams, leagues, or competitive levels. Recalibration was evaluated retrospectively, and no analysis demonstrates that model-assisted review improves athlete outcomes, communication, or decision quality in practice.

### Future research

Future research should test prospective validation with pre-specified outcomes, thresholds, recalibration procedures, and staff-review workflows. Independent-team validation is needed to evaluate transportability beyond SoccerMon. Studies should examine whether expanding-history or training-period-only SD definitions perform reliably when implemented in real time, whether team-specific recalibration is required, and how often thresholds should be updated.

Future applied studies should also evaluate human factors. Monitoring models may affect athlete trust, questionnaire behavior, staff workload, and communication. Prospective work should therefore assess not only discrimination and calibration but also whether model outputs improve the timeliness, quality, and fairness of staff review without replacing professional judgment or athlete-centered communication.

## Conclusions

This retrospective framework suggests that routine readiness, workload, and wellness data can provide incremental predictive information for prioritizing staff review of self-reported next-day readiness decline in elite female soccer. It is not an automated decision tool and has not demonstrated improved athlete management, performance, or outcomes. Prospective implementation studies and independent external validation in other teams are required before real-world application claims can be made.

## Supporting information

S1 File Supplementary methods and tables. This file contains supplementary methods for predictor construction, athlete-specific readiness-SD definitions, athlete-grouped out-of-fold threshold selection, calibration and Platt recalibration, athlete-level bootstrap uncertainty, and robustness analyses. It also includes Supplementary Tables S1–S17, covering the ordered predictor list, missingness and monitoring completeness, SD-definition sensitivity analyses, primary operating-threshold performance, calibration metrics, reliability-bin results, bootstrap uncertainty, athlete-grouped validation, internal cross-team robustness, top-risk capture comparison, standardized coefficients, domain-level coefficient contribution, descriptive subgroup calibration, outcome availability and exclusion patterns, readiness-decline magnitude sensitivity, regularization and coefficient-direction stability, and random-forest comparator sensitivity.
(DOCX)
