# Supplementary material for: Prioritizing next-day readiness decline in elite female soccer using readiness, workload, and wellness data: A temporally validated and calibrated monitoring framework
Source: PLoS One. 2026 Sep 10;21(9):e0358036. doi: 10.1371/journal.pone.0358036 (PMC13561368; doi:10.1371/journal.pone.0358036)
Supplement: Supplementary file 1 [file pone.0358036.s001.docx]

# Supplementary Material

**Contents**

Supplementary Methods 1–6

Supplementary Tables S1–S17

## Supplementary Methods 1. Predictor construction and nested model sets

Predictors were assembled into nested sets that sequentially added index-day readiness, workload magnitude, workload variability, workload instability, and wellness. Model 4 contained the prespecified 55 ordered predictors. Median imputation and standardization were fitted in the 2020 development data only and then applied unchanged to held-out 2021 data.

## Supplementary Methods 2. Athlete-specific readiness-SD definitions

The primary outcome was same-athlete next-calendar-day self-reported readiness decline of at least 0.5 athlete-specific SD under the expanding-history min5 definition. For each index date, the SD used only observed readiness values strictly before that date; index-day, next-day, and future readiness values were excluded. At least five prior observations and a positive defined historical SD were required; outcomes were not imputed. Full-period, fixed-2020, expanding-history min2, and expanding-history min7 definitions were sensitivity/reference analyses. Sensitivity analyses also used 0.25, 0.50, 0.75, and 1.00 SD outcome magnitudes. Availability mechanisms were not established and may reflect availability, participation, reporting behavior, or monitoring practice.

## Supplementary Methods 3. Athlete-grouped out-of-fold threshold selection

Models were trained on 2020 and evaluated on the held-out 2021 test set. The operating threshold was selected only from 2020 expanding-history min5 athlete-grouped out-of-fold (OOF) Model 4 predictions by balanced accuracy. The fixed primary threshold was 0.5011351162 (reported as 0.501) and was evaluated without use of 2021 labels for selection.

## Supplementary Methods 4. Calibration and Platt recalibration

Calibration summaries included Brier score, calibration-in-the-large, calibration intercept and slope, and bin-based ECE and MCE. Platt recalibration was fitted only on 2020 athlete-grouped OOF probabilities and applied once to fixed 2021 probabilities; it did not alter ranking, AUC, or PR-AUC. Descriptive subgroup calibration used these globally fixed Platt probabilities without subgroup-specific model fitting or recalibration.

## Supplementary Methods 5. Athlete-level bootstrap uncertainty

Uncertainty of fixed primary Model 4 2021 performance was estimated with 1,000 athlete-level bootstrap replicates, sampling athletes with replacement and retaining all sampled athlete-days. Paired athlete-level bootstrap uncertainty for Model 4 versus Model 0 used the same resampling unit. These analyses did not refit preprocessing, models, calibration, or thresholds.

## Supplementary Methods 6. Athlete-grouped and cross-team robustness analyses

Athlete-grouped validation retained each athlete in one fold. Internal cross-team holdout robustness trained on one SoccerMon team and tested on the other; it was not external validation. Prespecified L2 regularization and random-forest comparator sensitivities, coefficient-direction stability, and staff-review prioritization were evaluated as described in the corresponding tables. Standardized coefficients are model-dependent predictive summaries and are not causal effects.

## Supplementary Table S1. Ordered predictors in the 55-predictor Model 4.

| **Order** | **Predictor** | **Predictor domain** | **First model containing predictor** |
| --- | --- | --- | --- |
| 1 | acwr | Workload magnitude | Model 1 |
| 2 | atl | Workload magnitude | Model 1 |
| 3 | ctl28 | Workload magnitude | Model 1 |
| 4 | ctl42 | Workload magnitude | Model 1 |
| 5 | daily_load | Workload magnitude | Model 1 |
| 6 | daily_load_absolute_change | Workload instability | Model 3 |
| 7 | daily_load_roll14_cv | Workload variability | Model 2 |
| 8 | daily_load_roll14_mean | Workload magnitude | Model 1 |
| 9 | daily_load_roll14_std | Workload variability | Model 2 |
| 10 | daily_load_roll14_sum | Workload magnitude | Model 1 |
| 11 | daily_load_roll3_cv | Workload variability | Model 2 |
| 12 | daily_load_roll3_mean | Workload magnitude | Model 1 |
| 13 | daily_load_roll3_mean_absolute_change | Workload instability | Model 3 |
| 14 | daily_load_roll3_std | Workload variability | Model 2 |
| 15 | daily_load_roll3_sum | Workload magnitude | Model 1 |
| 16 | daily_load_roll7_cv | Workload variability | Model 2 |
| 17 | daily_load_roll7_mean | Workload magnitude | Model 1 |
| 18 | daily_load_roll7_std | Workload variability | Model 2 |
| 19 | daily_load_roll7_sum | Workload magnitude | Model 1 |
| 20 | fatigue | Wellness | Model 4 |
| 21 | fatigue_roll3_mean | Wellness | Model 4 |
| 22 | fatigue_roll3_std | Wellness | Model 4 |
| 23 | fatigue_roll7_mean | Wellness | Model 4 |
| 24 | fatigue_roll7_std | Wellness | Model 4 |
| 25 | load_7_28_ratio | Workload magnitude | Model 1 |
| 26 | monotony | Workload variability | Model 2 |
| 27 | mood | Wellness | Model 4 |
| 28 | mood_roll3_mean | Wellness | Model 4 |
| 29 | mood_roll3_std | Wellness | Model 4 |
| 30 | mood_roll7_mean | Wellness | Model 4 |
| 31 | mood_roll7_std | Wellness | Model 4 |
| 32 | readiness | Readiness | Model 0 |
| 33 | session_srpe | Workload magnitude | Model 1 |
| 34 | sleep_duration | Wellness | Model 4 |
| 35 | sleep_duration_roll3_mean | Wellness | Model 4 |
| 36 | sleep_duration_roll3_std | Wellness | Model 4 |
| 37 | sleep_duration_roll7_mean | Wellness | Model 4 |
| 38 | sleep_duration_roll7_std | Wellness | Model 4 |
| 39 | sleep_quality | Wellness | Model 4 |
| 40 | sleep_quality_roll3_mean | Wellness | Model 4 |
| 41 | sleep_quality_roll3_std | Wellness | Model 4 |
| 42 | sleep_quality_roll7_mean | Wellness | Model 4 |
| 43 | sleep_quality_roll7_std | Wellness | Model 4 |
| 44 | soreness | Wellness | Model 4 |
| 45 | soreness_roll3_mean | Wellness | Model 4 |
| 46 | soreness_roll3_std | Wellness | Model 4 |
| 47 | soreness_roll7_mean | Wellness | Model 4 |
| 48 | soreness_roll7_std | Wellness | Model 4 |
| 49 | strain | Workload variability | Model 2 |
| 50 | stress | Wellness | Model 4 |
| 51 | stress_roll3_mean | Wellness | Model 4 |
| 52 | stress_roll3_std | Wellness | Model 4 |
| 53 | stress_roll7_mean | Wellness | Model 4 |
| 54 | stress_roll7_std | Wellness | Model 4 |
| 55 | weekly_load | Workload magnitude | Model 1 |

Predictors are shown in the ordered sequence used by Model 4. Model 0 contained readiness only; subsequent models sequentially added workload magnitude, workload variability, workload instability, and wellness predictors.

## Supplementary Table S2. Predictor missingness and monitoring completeness.

**Panel A. Predictor-level missingness**

| **Predictor** | **Predictor domain** | **Available observations** | **Missing observations** | **Missing (%)** |
| --- | --- | --- | --- | --- |
| acwr | Workload magnitude | 14,428 | 0 | 0.000 |
| atl | Workload magnitude | 14,428 | 0 | 0.000 |
| ctl28 | Workload magnitude | 14,428 | 0 | 0.000 |
| ctl42 | Workload magnitude | 14,428 | 0 | 0.000 |
| daily_load | Workload magnitude | 14,428 | 0 | 0.000 |
| daily_load_absolute_change | Workload instability | 14,428 | 0 | 0.000 |
| daily_load_roll14_cv | Workload variability | 14,357 | 71 | 0.492 |
| daily_load_roll14_mean | Workload magnitude | 14,428 | 0 | 0.000 |
| daily_load_roll14_std | Workload variability | 14,428 | 0 | 0.000 |
| daily_load_roll14_sum | Workload magnitude | 14,428 | 0 | 0.000 |
| daily_load_roll3_cv | Workload variability | 13,832 | 596 | 4.131 |
| daily_load_roll3_mean | Workload magnitude | 14,428 | 0 | 0.000 |
| daily_load_roll3_mean_absolute_change | Workload instability | 14,428 | 0 | 0.000 |
| daily_load_roll3_std | Workload variability | 14,428 | 0 | 0.000 |
| daily_load_roll3_sum | Workload magnitude | 14,428 | 0 | 0.000 |
| daily_load_roll7_cv | Workload variability | 14,240 | 188 | 1.303 |
| daily_load_roll7_mean | Workload magnitude | 14,428 | 0 | 0.000 |
| daily_load_roll7_std | Workload variability | 14,428 | 0 | 0.000 |
| daily_load_roll7_sum | Workload magnitude | 14,428 | 0 | 0.000 |
| fatigue | Wellness | 14,421 | 7 | 0.049 |
| fatigue_roll3_mean | Wellness | 13,957 | 471 | 3.264 |
| fatigue_roll3_std | Wellness | 13,957 | 471 | 3.264 |
| fatigue_roll7_mean | Wellness | 14,146 | 282 | 1.955 |
| fatigue_roll7_std | Wellness | 14,146 | 282 | 1.955 |
| load_7_28_ratio | Workload magnitude | 14,406 | 22 | 0.152 |
| monotony | Workload variability | 14,428 | 0 | 0.000 |
| mood | Wellness | 14,423 | 5 | 0.035 |
| mood_roll3_mean | Wellness | 13,959 | 469 | 3.251 |
| mood_roll3_std | Wellness | 13,959 | 469 | 3.251 |
| mood_roll7_mean | Wellness | 14,146 | 282 | 1.955 |
| mood_roll7_std | Wellness | 14,146 | 282 | 1.955 |
| readiness | Readiness | 14,428 | 0 | 0.000 |
| session_srpe | Workload magnitude | 10,819 | 3,609 | 25.014 |
| sleep_duration | Wellness | 14,407 | 21 | 0.146 |
| sleep_duration_roll3_mean | Wellness | 13,957 | 471 | 3.264 |
| sleep_duration_roll3_std | Wellness | 13,957 | 471 | 3.264 |
| sleep_duration_roll7_mean | Wellness | 14,146 | 282 | 1.955 |
| sleep_duration_roll7_std | Wellness | 14,146 | 282 | 1.955 |
| sleep_quality | Wellness | 14,417 | 11 | 0.076 |
| sleep_quality_roll3_mean | Wellness | 13,962 | 466 | 3.230 |
| sleep_quality_roll3_std | Wellness | 13,962 | 466 | 3.230 |
| sleep_quality_roll7_mean | Wellness | 14,147 | 281 | 1.948 |
| sleep_quality_roll7_std | Wellness | 14,147 | 281 | 1.948 |
| soreness | Wellness | 14,424 | 4 | 0.028 |
| soreness_roll3_mean | Wellness | 13,960 | 468 | 3.244 |
| soreness_roll3_std | Wellness | 13,960 | 468 | 3.244 |
| soreness_roll7_mean | Wellness | 14,147 | 281 | 1.948 |
| soreness_roll7_std | Wellness | 14,147 | 281 | 1.948 |
| strain | Workload variability | 14,428 | 0 | 0.000 |
| stress | Wellness | 14,421 | 7 | 0.049 |
| stress_roll3_mean | Wellness | 13,962 | 466 | 3.230 |
| stress_roll3_std | Wellness | 13,962 | 466 | 3.230 |
| stress_roll7_mean | Wellness | 14,145 | 283 | 1.961 |
| stress_roll7_std | Wellness | 14,145 | 283 | 1.961 |
| weekly_load | Workload magnitude | 14,428 | 0 | 0.000 |

Predictor missingness was assessed in the locked expanding-history min5 primary analytical dataset. Missing (%) = missing observations divided by 14,428 eligible athlete-days × 100. Outcomes were not imputed; predictor median imputation was fitted only within training data or a validation fold.

**Panel B. Athlete-level monitoring completeness**

| **Athlete** | **Eligible athlete-days** | **Readiness-decline events** | **Event rate** | **Valid min5 pairs** |
| --- | --- | --- | --- | --- |
| Athlete 01 | 182 | 45 | 0.247 | 182 |
| Athlete 02 | 520 | 112 | 0.215 | 520 |
| Athlete 03 | 424 | 145 | 0.342 | 424 |
| Athlete 04 | 337 | 76 | 0.226 | 337 |
| Athlete 05 | 184 | 54 | 0.293 | 184 |
| Athlete 06 | 544 | 121 | 0.222 | 544 |
| Athlete 07 | 271 | 85 | 0.314 | 271 |
| Athlete 08 | 609 | 164 | 0.269 | 609 |
| Athlete 09 | 407 | 98 | 0.241 | 407 |
| Athlete 10 | 628 | 90 | 0.143 | 628 |
| Athlete 11 | 232 | 44 | 0.190 | 232 |
| Athlete 12 | 269 | 98 | 0.364 | 269 |
| Athlete 13 | 634 | 104 | 0.164 | 634 |
| Athlete 14 | 261 | 62 | 0.238 | 261 |
| Athlete 15 | 112 | 28 | 0.250 | 112 |
| Athlete 16 | 336 | 121 | 0.360 | 336 |
| Athlete 17 | 226 | 42 | 0.186 | 226 |
| Athlete 18 | 434 | 88 | 0.203 | 434 |
| Athlete 19 | 288 | 91 | 0.316 | 288 |
| Athlete 20 | 579 | 132 | 0.228 | 579 |
| Athlete 21 | 326 | 94 | 0.288 | 326 |
| Athlete 22 | 157 | 54 | 0.344 | 157 |
| Athlete 23 | 85 | 22 | 0.259 | 85 |
| Athlete 24 | 337 | 87 | 0.258 | 337 |
| Athlete 25 | 216 | 55 | 0.255 | 216 |
| Athlete 26 | 610 | 150 | 0.246 | 610 |
| Athlete 27 | 395 | 123 | 0.311 | 395 |
| Athlete 28 | 65 | 17 | 0.262 | 65 |
| Athlete 29 | 132 | 33 | 0.250 | 132 |
| Athlete 30 | 405 | 113 | 0.279 | 405 |
| Athlete 31 | 384 | 51 | 0.133 | 384 |
| Athlete 32 | 512 | 13 | 0.025 | 512 |
| Athlete 33 | 53 | 3 | 0.057 | 53 |
| Athlete 34 | 98 | 29 | 0.296 | 98 |
| Athlete 35 | 49 | 12 | 0.245 | 49 |
| Athlete 36 | 85 | 16 | 0.188 | 85 |
| Athlete 37 | 89 | 18 | 0.202 | 89 |
| Athlete 38 | 478 | 105 | 0.220 | 478 |
| Athlete 39 | 483 | 100 | 0.207 | 483 |
| Athlete 40 | 70 | 20 | 0.286 | 70 |
| Athlete 41 | 298 | 91 | 0.305 | 298 |
| Athlete 42 | 3 | 0 | 0.000 | 3 |
| Athlete 43 | 70 | 18 | 0.257 | 70 |
| Athlete 44 | 357 | 88 | 0.246 | 357 |
| Athlete 45 | 194 | 53 | 0.273 | 194 |
| Athlete 46 | 399 | 115 | 0.288 | 399 |
| Athlete 47 | 26 | 7 | 0.269 | 26 |
| Athlete 48 | 18 | 7 | 0.389 | 18 |
| Athlete 49 | 489 | 78 | 0.160 | 489 |
| Athlete 50 | 68 | 18 | 0.265 | 68 |

Athlete-level values are descriptive summaries of the locked min5 eligible dataset. Missingness mechanisms could not be established and may reflect availability, participation, reporting behavior, or monitoring practice.

## Supplementary Table S3. Detailed sensitivity analysis across athlete-specific readiness-SD definitions.

| **SD definition** | **Analytical role** | **Athletes** | **Eligible athlete-days** | **Events** | **Event rate** | **Rows excluded vs full-period** | **AUC** | **PR-AUC** | **Brier** |
| --- | --- | --- | --- | --- | --- | --- | --- | --- | --- |
| Expanding-history min5 | Primary analysis | 50 | 14,428 | 3,390 | 0.235 | 203 | 0.7645 | 0.5135 | 0.1924 |
| Full-period SD | Retrospective sensitivity/reference | 50 | 14,631 | 3,462 | 0.237 | 0 | 0.7660 | 0.5209 | 0.1901 |
| Fixed-2020 SD | Sensitivity | 37 | 13,231 | 3,114 | 0.235 | 1400 | 0.7682 | 0.5208 | 0.1829 |
| Expanding-history min2 | Sensitivity | 50 | 14,516 | 3,420 | 0.236 | 115 | 0.7642 | 0.5134 | 0.1926 |
| Expanding-history min7 | Sensitivity | 50 | 14,347 | 3,362 | 0.234 | 284 | 0.7630 | 0.5112 | 0.1934 |

Expanding-history min5 is the primary outcome definition. Full-period SD is retrospective sensitivity/reference only. AUC, PR-AUC, and Brier score are threshold-independent performance measures.

## Supplementary Table S4. Primary athlete-grouped out-of-fold operating threshold and held-out 2021 performance.

| **Threshold-selection method** | **Threshold** | **Sensitivity** | **Specificity** | **Balanced accuracy** | **F1** | **Predicted-positive rate** | **AUC** | **PR-AUC** | **Brier** |
| --- | --- | --- | --- | --- | --- | --- | --- | --- | --- |
| 2020 athlete-grouped OOF balanced-accuracy selection (primary) | 0.501 | 0.687 | 0.704 | 0.695 | 0.512 | 0.386 | 0.7645 | 0.5135 | 0.1924 |

The primary threshold was selected only from 2020 expanding-history min5 athlete-grouped OOF predictions by balanced accuracy and applied unchanged to the held-out 2021 test set.

## Supplementary Table S5. Calibration metrics before and after Platt recalibration.

| **Metric** | **Uncalibrated** | **Platt recalibrated** |
| --- | --- | --- |
| Observed prevalence | 0.2287 | 0.2287 |
| Mean predicted probability | 0.4254 | 0.2334 |
| Brier score | 0.1924 | 0.1460 |
| Constant-risk Brier reference | 0.1764 | 0.1764 |
| Brier Skill Score | -0.0905 | 0.1725 |
| Calibration-in-the-large | -1.1686 | -0.0307 |
| Calibration intercept | -1.1493 | 0.0765 |
| Calibration slope | 0.9002 | 1.1098 |
| ECE | 0.1967 | 0.0226 |
| MCE | 0.3268 | 0.0721 |
| AUC | 0.7645 | 0.7645 |
| PR-AUC | 0.5135 | 0.5135 |

Calibration-in-the-large was estimated using an offset logistic model; calibration intercept and slope came from a freely estimated model. Platt recalibration was unweighted, fitted only on 2020 grouped-OOF predictions, and applied once to untouched 2021 predictions.

## Supplementary Table S6. Reliability-bin results for uncalibrated and Platt-recalibrated probabilities.

| **Calibration state** | **Bin** | **Row count** | **Mean predicted probability** | **Observed event proportion** | **Absolute calibration error** |
| --- | --- | --- | --- | --- | --- |
| Uncalibrated | 1 | 836 | 0.0697 | 0.0514 | 0.0182 |
| Uncalibrated | 2 | 836 | 0.1503 | 0.0778 | 0.0725 |
| Uncalibrated | 3 | 835 | 0.2227 | 0.0826 | 0.1400 |
| Uncalibrated | 4 | 836 | 0.2946 | 0.1112 | 0.1834 |
| Uncalibrated | 5 | 835 | 0.3702 | 0.1593 | 0.2109 |
| Uncalibrated | 6 | 836 | 0.4490 | 0.1938 | 0.2552 |
| Uncalibrated | 7 | 835 | 0.5294 | 0.2671 | 0.2623 |
| Uncalibrated | 8 | 836 | 0.6163 | 0.2895 | 0.3268 |
| Uncalibrated | 9 | 835 | 0.7111 | 0.4108 | 0.3003 |
| Uncalibrated | 10 | 836 | 0.8407 | 0.6435 | 0.1971 |
| Platt recalibrated | 1 | 836 | 0.0385 | 0.0514 | 0.0129 |
| Platt recalibrated | 2 | 836 | 0.0752 | 0.0778 | 0.0026 |
| Platt recalibrated | 3 | 835 | 0.1073 | 0.0826 | 0.0247 |
| Platt recalibrated | 4 | 836 | 0.1404 | 0.1112 | 0.0292 |
| Platt recalibrated | 5 | 835 | 0.1773 | 0.1593 | 0.0180 |
| Platt recalibrated | 6 | 836 | 0.2193 | 0.1938 | 0.0256 |
| Platt recalibrated | 7 | 835 | 0.2675 | 0.2671 | 0.0004 |
| Platt recalibrated | 8 | 836 | 0.3279 | 0.2895 | 0.0384 |
| Platt recalibrated | 9 | 835 | 0.4088 | 0.4108 | 0.0019 |
| Platt recalibrated | 10 | 836 | 0.5714 | 0.6435 | 0.0721 |

Reliability bins were constructed from the fixed held-out 2021 expanding-history min5 predictions using the same quantile-binning procedure as the calibration analysis in Fig 3. Platt probabilities used the globally fitted 2020 athlete-grouped OOF recalibration model; no model or calibration refitting was performed within bins.

## Supplementary Table S7. Athlete-level bootstrap uncertainty for the primary Model 4 result.

| **Panel** | **Metric** | **Point estimate** | **Lower 95% CI** | **Upper 95% CI** | **Valid replicates** |
| --- | --- | --- | --- | --- | --- |
| Panel A: Model 4 performance | AUC | 0.764475 | 0.729450 | 0.796205 | 1,000 |
| Panel A: Model 4 performance | PR-AUC | 0.513489 | 0.458628 | 0.571257 | 1,000 |
| Panel A: Model 4 performance | Brier | 0.192351 | 0.173365 | 0.211601 | 1,000 |
| Panel B: Paired increment | ΔAUC (Model 4 − Model 0) | 0.0592 | 0.0288 | 0.0891 | 1,000 |
| Panel B: Paired increment | ΔPR-AUC (Model 4 − Model 0) | 0.1296 | 0.0897 | 0.1677 | 1,000 |
| Panel B: Paired increment | Brier improvement (Model 0 − Model 4) | 0.0281 | 0.0141 | 0.0435 | 1,000 |

Panel A reports held-out primary Model 4 performance uncertainty conditional on fixed predictions; Panel B reports paired incremental uncertainty. Athletes, not rows, were sampled with replacement in 1,000 replicates.

## Supplementary Table S8. Athlete-grouped validation of the expanding-history min5 Model 4 logistic regression.

| **Fold** | **Training rows** | **Test rows** | **Threshold** | **AUC** | **PR-AUC** | **Brier** | **Sensitivity** | **Specificity** | **Balanced accuracy** | **F1** | **Predicted-positive rate** |
| --- | --- | --- | --- | --- | --- | --- | --- | --- | --- | --- | --- |
| Fold 1 | 11410 | 3018 | 0.510 | 0.734 | 0.495 | 0.233 | 0.741 | 0.577 | 0.659 | 0.459 | 0.493 |
| Fold 2 | 11604 | 2824 | 0.520 | 0.799 | 0.592 | 0.176 | 0.665 | 0.782 | 0.723 | 0.567 | 0.327 |
| Fold 3 | 11491 | 2937 | 0.511 | 0.764 | 0.533 | 0.229 | 0.767 | 0.582 | 0.675 | 0.480 | 0.497 |
| Fold 4 | 11555 | 2873 | 0.495 | 0.784 | 0.533 | 0.173 | 0.642 | 0.770 | 0.706 | 0.537 | 0.327 |
| Fold 5 | 11652 | 2776 | 0.557 | 0.749 | 0.497 | 0.189 | 0.524 | 0.813 | 0.668 | 0.502 | 0.272 |
| Mean | 11542 | 2886 | 0.519 | 0.766 | 0.530 | 0.200 | 0.668 | 0.705 | 0.686 | 0.509 | 0.383 |

Rows show fold-level athlete-grouped validation for the Model 4 logistic regression under the expanding-history min5 definition. This was not validation in an independent cohort.

## Supplementary Table S9. Internal cross-team holdout robustness analysis within SoccerMon.

| **Train team** | **Test team** | **Training rows** | **Test rows** | **Threshold** | **AUC** | **PR-AUC** | **Brier** | **Sensitivity** | **Specificity** | **Balanced accuracy** | **F1** | **Predicted-positive rate** |
| --- | --- | --- | --- | --- | --- | --- | --- | --- | --- | --- | --- | --- |
| TeamA | TeamB | 9603 | 4825 | 0.538 | 0.757 | 0.469 | 0.235 | 0.751 | 0.619 | 0.685 | 0.470 | 0.458 |
| TeamB | TeamA | 4825 | 9603 | 0.500 | 0.748 | 0.506 | 0.208 | 0.691 | 0.668 | 0.680 | 0.513 | 0.421 |

This was an internal cross-team robustness analysis within SoccerMon and was not independent external validation.

## Supplementary Table S10. Top-risk capture comparison between Model 0 and Model 4

| **Model** | **Review proportion** | **Athlete-days reviewed** | **Events captured** | **Event capture rate** | **PPV** | **Lift vs test prevalence** | **Athlete-days/event captured** |
| --- | --- | --- | --- | --- | --- | --- | --- |
| Model 0 | 5% | 418 | 200 | 10.5% | 47.8% | 2.09 | 2.09 |
| Model 0 | 10% | 836 | 392 | 20.5% | 46.9% | 2.05 | 2.13 |
| Model 0 | 20% | 1,672 | 696 | 36.4% | 41.6% | 1.82 | 2.40 |
| Model 0 | 30% | 2,507 | 993 | 52.0% | 39.6% | 1.73 | 2.52 |
| Model 0 | 40% | 3,343 | 1,205 | 63.1% | 36.0% | 1.58 | 2.77 |
| Model 0 | 50% | 4,178 | 1,421 | 74.4% | 34.0% | 1.49 | 2.94 |
| Model 4 | 5% | 418 | 295 | 15.4% | 70.6% | 3.09 | 1.42 |
| Model 4 | 10% | 836 | 538 | 28.2% | 64.4% | 2.81 | 1.55 |
| Model 4 | 20% | 1,672 | 881 | 46.1% | 52.7% | 2.30 | 1.90 |
| Model 4 | 30% | 2,507 | 1,123 | 58.8% | 44.8% | 1.96 | 2.23 |
| Model 4 | 40% | 3,343 | 1,346 | 70.4% | 40.3% | 1.76 | 2.48 |
| Model 4 | 50% | 4,178 | 1,508 | 78.9% | 36.1% | 1.58 | 2.77 |

## Supplementary Table S11. Model 4 standardized coefficient ranking

| **Predictor** | **Domain** | **Standardized coefficient** | **Absolute coefficient** | **Direction** |
| --- | --- | --- | --- | --- |
| weekly_load | workload | -1.209 | 1.209 | negative |
| atl | workload | 0.919 | 0.919 | positive |
| readiness | readiness | 0.834 | 0.834 | positive |
| daily_load | workload | 0.653 | 0.653 | positive |
| fatigue_roll7_mean | wellness | -0.276 | 0.276 | negative |
| daily_load_roll3_std | workload | -0.186 | 0.186 | negative |
| daily_load_roll7_sum | workload | 0.179 | 0.179 | positive |
| daily_load_roll7_mean | workload | 0.179 | 0.179 | positive |
| daily_load_roll14_cv | workload | 0.161 | 0.161 | positive |
| daily_load_roll3_sum | workload | -0.151 | 0.151 | negative |
| daily_load_roll3_mean | workload | -0.151 | 0.151 | negative |
| ctl28 | workload | -0.131 | 0.131 | negative |
| session_srpe | workload | 0.130 | 0.130 | positive |
| fatigue | wellness | 0.128 | 0.128 | positive |
| fatigue_roll3_mean | wellness | 0.118 | 0.118 | positive |
| mood_roll3_mean | wellness | 0.118 | 0.118 | positive |
| stress_roll7_std | wellness | 0.117 | 0.117 | positive |
| daily_load_absolute_change | workload | 0.117 | 0.117 | positive |
| fatigue_roll7_std | wellness | 0.114 | 0.114 | positive |
| daily_load_roll3_cv | workload | -0.111 | 0.111 | negative |

Standardized coefficients are model-dependent predictive summaries and should not be interpreted causally because predictors are correlated and the model used class weighting.

## Supplementary Table S12. Domain-level coefficient contribution

| Domain | Sum absolute coefficients | Mean absolute coefficients | Number of features |
| --- | --- | --- | --- |
| readiness | 0.834 | 0.834 | 1 |
| wellness | 2.045 | 0.068 | 30 |
| workload_instability | 0.157 | 0.078 | 2 |
| workload_magnitude | 4.005 | 0.286 | 14 |
| workload_variability | 0.775 | 0.097 | 8 |

## Supplementary Table S13. Descriptive subgroup calibration by team and 2021 calendar month in the held-out primary analysis.

| **Panel** | **Subgroup** | **n** | **Events** | **Observed prevalence** | **Mean Platt probability** | **Brier** | **Calibration-in-the-large** |
| --- | --- | --- | --- | --- | --- | --- | --- |
| Team | TeamA | 5,891 | 1,424 | 0.2417 | 0.2325 | 0.1516 | 0.0602 |
| Team | TeamB | 2,465 | 487 | 0.1976 | 0.2355 | 0.1325 | -0.2563 |
| Calendar month | 2021-01 | 675 | 185 | 0.2741 | 0.2746 | 0.1605 | -0.0031 |
| Calendar month | 2021-02 | 734 | 176 | 0.2398 | 0.2374 | 0.1430 | 0.0151 |
| Calendar month | 2021-03 | 829 | 212 | 0.2557 | 0.2469 | 0.1614 | 0.0546 |
| Calendar month | 2021-04 | 624 | 128 | 0.2051 | 0.2314 | 0.1329 | -0.1772 |
| Calendar month | 2021-05 | 710 | 143 | 0.2014 | 0.2209 | 0.1258 | -0.1367 |
| Calendar month | 2021-06 | 676 | 146 | 0.2160 | 0.2318 | 0.1381 | -0.1045 |
| Calendar month | 2021-07 | 662 | 132 | 0.1994 | 0.2319 | 0.1307 | -0.2199 |
| Calendar month | 2021-08 | 951 | 212 | 0.2229 | 0.2424 | 0.1437 | -0.1270 |
| Calendar month | 2021-09 | 878 | 192 | 0.2187 | 0.2195 | 0.1364 | -0.0057 |
| Calendar month | 2021-10 | 832 | 197 | 0.2368 | 0.2116 | 0.1530 | 0.1692 |
| Calendar month | 2021-11 | 473 | 107 | 0.2262 | 0.2108 | 0.1596 | 0.1065 |
| Calendar month | 2021-12 | 312 | 81 | 0.2596 | 0.2411 | 0.1964 | 0.1204 |

## Supplementary Table S14. Outcome availability and exclusion pattern for the expanding-history min5 primary analysis. Panel A summarizes sequential exclusions overall and by year and team. Panel B summarizes athlete-level variation in next-day outcome availability and final eligibility. All summaries are descriptive; missingness mechanisms were not established and may reflect availability, participation, reporting behavior, or monitoring practice.

| **Panel** | **Stage or stratum** | **Rows** | **Eligible / candidate (%)** |
| --- | --- | --- | --- |
| Overall | Source daily rows | 36,550 | 100.00% |
| Overall | Eligible primary rows | 14,428 | 39.47% |
| Overall | missing or invalid athlete id or date | 0 |  |
| Overall | missing index day readiness | 19,553 |  |
| Overall | no same athlete next calendar day observation | 11 |  |
| Overall | missing next day readiness | 2,355 |  |
| Overall | insufficient prior history under 5 | 201 |  |
| Overall | nonpositive or undefined prior sd | 2 |  |
| year | 2020 | 6,072 | 33.18% |
| year | 2021 | 8,356 | 45.79% |
| team | TeamA | 9,603 | 48.65% |
| team | TeamB | 4,825 | 28.70% |

Panel B. Athlete-level distribution of next-day outcome availability and final primary eligibility (n=50).

| Measure | Minimum | Q1 | Median | Q3 | Maximum |
| --- | --- | --- | --- | --- | --- |
| Next-day readiness observed (% of source rows) | 0.7% | 14.3% | 38.9% | 58.1% | 87.4% |
| Next-day readiness unavailable (% of same-athlete next-day candidate rows) | 0.9% | 6.5% | 15.6% | 26.8% | 78.3% |
| Final primary eligibility (% of source rows) | 0.4% | 13.9% | 38.2% | 57.4% | 86.7% |

Eligibility is descriptive. Missingness mechanisms were not established and may reflect availability, participation, reporting behavior, or monitoring practice.

## Supplementary Table S15. Sensitivity to alternative magnitudes used to define next-day readiness decline.

| **Threshold** | **Eligible athlete-days** | **Events** | **Prevalence** | **Model 0 AUC** | **Model 4 AUC** | **ΔAUC** | **Model 0 PR-AUC** | **Model 4 PR-AUC** | **ΔPR-AUC** | **Model 4 Brier** |
| --- | --- | --- | --- | --- | --- | --- | --- | --- | --- | --- |
| 0.25 SD | 14,428 | 3,407 | 0.236 | 0.7052 | 0.7660 | 0.0608 | 0.3833 | 0.5178 | 0.1345 | 0.1918 |
| 0.50 SD | 14,428 | 3,390 | 0.235 | 0.7043 | 0.7645 | 0.0601 | 0.3808 | 0.5135 | 0.1326 | 0.1924 |
| 0.75 SD | 14,428 | 3,031 | 0.210 | 0.7166 | 0.7734 | 0.0568 | 0.3500 | 0.4903 | 0.1403 | 0.1873 |
| 1.00 SD | 14,428 | 2,321 | 0.161 | 0.7061 | 0.7565 | 0.0504 | 0.2891 | 0.3859 | 0.0967 | 0.1874 |

The magnitude thresholds are athlete-relative operational definitions and are not clinically or physiologically validated cutoffs.

## Supplementary Table S16. L2 regularization sensitivity and leading coefficient-direction stability.

| Panel | Specification / predictor | Primary coefficient | AUC | PR-AUC | Brier | Fold direction consistency |
| --- | --- | --- | --- | --- | --- | --- |
| Panel A | C=0.1 |  | 0.7652 | 0.5141 | 0.1935 |  |
| Panel A | C=0.3 |  | 0.7650 | 0.5140 | 0.1930 |  |
| Panel A | C=1.0 |  | 0.7645 | 0.5135 | 0.1924 |  |
| Panel A | C=3.0 |  | 0.7619 | 0.5102 | 0.1926 |  |
| Panel B | weekly_load | -1.209 |  |  |  | 80% negative |
| Panel B | atl | 0.919 |  |  |  | 100% positive |
| Panel B | readiness | 0.834 |  |  |  | 100% positive |
| Panel B | daily_load | 0.653 |  |  |  | 100% positive |
| Panel B | fatigue_roll7_mean | -0.276 |  |  |  | 100% negative |

C=1.0 was prespecified; no configuration was selected using 2021 performance. Coefficients are predictive summaries, not causal effects.

## Supplementary Table S17. Random-forest comparator sensitivity in the held-out 2021 test set.

| **Configuration** | **n_estimators** | **max_depth** | **min_samples_leaf** | **AUC** | **PR-AUC** | **Brier** |
| --- | --- | --- | --- | --- | --- | --- |
| RF-1 | 250 | 5 | 10 | 0.7470 | 0.4866 | 0.2033 |
| RF-2 | 500 | 5 | 10 | 0.7472 | 0.4881 | 0.2032 |
| RF-3 | 250 | 10 | 10 | 0.7535 | 0.5026 | 0.1774 |
| RF-4 | 500 | 10 | 10 | 0.7538 | 0.5030 | 0.1775 |
| RF-5 | 250 | None | 10 | 0.7532 | 0.4996 | 0.1721 |
| RF-6 | 500 | None | 10 | 0.7540 | 0.5042 | 0.1720 |

Logistic regression retained higher discrimination; some deeper random-forest specifications had lower raw uncalibrated Brier. The logistic model remained primary for transparency, auditability, coefficient interpretation, and the prespecified calibration framework.
